# Genome comparison between clinical and environmental strains of *Herbaspirillum seropedicae* reveals a potential new emerging bacterium adapted to human hosts

**DOI:** 10.1186/s12864-019-5982-9

**Published:** 2019-08-02

**Authors:** Helisson Faoro, Willian K. Oliveira, Vinicius A. Weiss, Michelle Z. Tadra-Sfeir, Rodrigo L. Cardoso, Eduardo Balsanelli, Liziane C. C. Brusamarello-Santos, Doumit Camilios-Neto, Leonardo M. Cruz, Roberto T. Raittz, Ana C. Q. Marques, John LiPuma, Cyntia M. T. Fadel-Picheth, Emanuel M. Souza, Fabio O. Pedrosa

**Affiliations:** 1 Department of Biochemistry and Molecular Biology, Universidade Federal do Paraná, Coronel Francisco H. dos Santos street, Curitiba, Paraná 81531-980 Brazil; 2 Graduate Program on Bioinformatics, Universidade Federal do Paraná, Alcides Viera Arcoverde street 1225, Curitiba, Paraná 81520-260 Brazil; 3 Laboratory of Gene Expression Regulation, Instituto Carlos Chagas, FIOCRUZ, Algacyr Munhoz Mader street, 3775, Curitiba, Paraná 81350-010 Brazil; 4 Department of Clinical Analyses, Universidade Federal do Paraná, Av. Lothário Meissner 632, Curitiba, Paraná 80210-170 Brazil; 5 Department of Biochemistry and Biothecnology, Universidade Estadual de Londrina, Celso Garcia Cid street, Londrina, Paraná 86057-970 Brazil; 6 Department of Pediatrics, University of Michigan, 1500 E. Medical Center Dr, Ann Arbor, MI 48109 USA

**Keywords:** *Herbaspirillum seropedicae*, Clinical isolates, Genome comparison, Genomic adaptation, LPS substitution

## Abstract

**Background:**

*Herbaspirillum seropedicae* is an environmental β-proteobacterium that is capable of promoting the growth of economically relevant plants through biological nitrogen fixation and phytohormone production. However, strains of *H. seropedicae* have been isolated from immunocompromised patients and associated with human infections and deaths. In this work, we sequenced the genomes of two clinical strains of *H. seropedicae*, AU14040 and AU13965, and compared them with the genomes of strains described as having an environmental origin.

**Results:**

Both genomes were closed, indicating a single circular chromosome; however, strain AU13965 also carried a plasmid of 42,977 bp, the first described in the genus *Herbaspirillum*. Genome comparison revealed that the clinical strains lost the gene sets related to biological nitrogen fixation (*nif*) and the type 3 secretion system (T3SS), which has been described to be essential for interactions with plants. Comparison of the pan-genomes of clinical and environmental strains revealed different sets of accessorial genes. However, antimicrobial resistance genes were found in the same proportion in all analyzed genomes. The clinical strains also acquired new genes and genomic islands that may be related to host interactions. Among the acquired islands was a cluster of genes related to lipopolysaccharide (LPS) biosynthesis. Although highly conserved in environmental strains, the LPS biosynthesis genes in the two clinical strains presented unique and non-orthologous genes within the genus *Herbaspirillum*. Furthermore, the AU14040 strain cluster contained the *neuABC* genes, which are responsible for sialic acid (Neu5Ac) biosynthesis, indicating that this bacterium could add it to its lipopolysaccharide. The Neu5Ac-linked LPS could increase the bacterial resilience in the host aiding in the evasion of the immune system.

**Conclusions:**

Our findings suggest that the lifestyle transition from environment to opportunist led to the loss and acquisition of specific genes allowing adaptations to colonize and survive in new hosts. It is possible that these substitutions may be the starting point for interactions with new hosts.

**Electronic supplementary material:**

The online version of this article (10.1186/s12864-019-5982-9) contains supplementary material, which is available to authorized users.

## Background

The genus *Herbaspirillum* belongs to the class β-Proteobacteria and has 11 described species. These species were isolated from diverse environments such as water, contaminated soil, plant rhizosphere, plant internal tissues and root nodules. The first described species of the genus *Herbaspirillum* was the bacterium *Herbaspirillum seropedicae*, which was found colonizing the roots and aerial parts of important crops such as rice, sugarcane, maize and also tropical species such as pineapple and banana [1–3]. Since its isolation, this species has been verified as an endophytic non-pathogenic bacterium. It is able to promote plant growth through the production of phytohormones and by performing biological nitrogen fixation, a process through which diazotrophic bacteria convert atmospheric nitrogen (N_2_) to ammonium (NH^4+^) [4, 5]. Sequencing and analysis of the *H. seropedicae* SmR1 genome allowed identification of the genes involved in nitrogen fixation (*nif*), genes related to nitrate metabolism (*nar*, *nas*, *nir*), plant ethylene stress-relieving 1-aminocyclopropane-1-carboxylate deaminase (*accD*) and the plant-bacteria interaction type three secretion system (T3SS) [6].

The first description of a *Herbaspirillum* genus bacterium isolated from clinical samples occurred in 1996 [7]. In this work, Baldani and coworkers analyzed the phenotypes of 147 isolates and verified that a group of strains, previously denominated as “group EF1”, belonged to the genus *Herbaspirillum*. However, the low degree of DNA-DNA hybridization (~ 50%) did not allow for the identification of any of the *Herbaspirillum* species described at that time, and these strains were tentatively referred to as *Herbaspirillum* species 3 [7]. This group of bacteria was isolated from different types of human samples, such as samples from wounds, urine, feces, bacteremia, and gastritis as well as pulmonary, ocular and pharyngeal infections. Since then, new species of the genus *Herbaspirillum* have been associated with clinical samples. Swantarat et al. reported the first fatal case-related *H. seropedicae* infection: a case of bacteremia secondary to pneumonia in a 65-year-old man with end-stage renal disease and multiple myeloma [8]. Chemically et al. (2015) reported a study conducted at the University of Texas MD Anderson Cancer Center in which cases of patients with sepsis, whose infectious agents were previously classified as *Burkholderia* sp., were actually bacteria of the genus *Herbaspirillum* [9]. A similar case was described at the University of Michigan Pediatric Hospital where a reanalysis indicated that of 1,100 bacteria isolated from the sputum samples of patients with cystic fibrosis, previously classified as *Burkholderia* sp., approximately 3% (28 isolates) were from the *Herbaspirillum* genus [10]. The isolates from this study were classified by 16S rRNA gene sequence analysis as *H. seropedicae* (2 isolates), *Herbaspirillum huttiense* sp. *putei* (2 isolates), *Herbaspirillum huttiense* sp. *huttiense* (3 isolates), and *Herbaspirillum frisingense* (3 isolates). In addition, 18 isolates that could not be classified were named lineages 1, 2 and 3 [10]. Comparison of *Herbaspirillum* isolates recovered from environmental and clinical sources showed no differences in adhesion capacity and cytotoxicity in human HeLa cells [11]. Although the above cases are related to immunocompromised patients, Regunath et al. described a case of bacteremia caused by gentamicin-resistant *Herbaspirillum* in an immunocompetent adult male farmer [12].

Sequencing and comparison of genomes has been a powerful tool for phylogenetic studies and has helped elucidate the different characteristics of environmental and opportunistic (or pathogenic) bacteria within the same taxonomic group [13, 14]. Thus, to better understand how these bacteria from the *Herbaspirillum* genus are migrating from the environment to human hosts, we sequenced the genomes of two clinical isolates, identified as *Herbaspirillum seropedicae* AU14040 and *Herbaspirillum* lineage 2 AU13965, isolated from cystic fibrosis patients by Spilker and collaborators [10]. We compared these clinical isolate genomes with the genomes of environmental isolates of *H. seropedicae* species and revealed gene losses and acquisitions that provide evidence for how strains of this species may evolve from plant endophytes to opportunistic human pathogens.

## Results

### Genome assembly

Genome assembly was performed through a hybrid strategy using data from different sequencing platforms, different assembly programs and gap closure. The initial *H. seropedicae* AU14040 genome assembly had 52 contigs in 15 scaffolds. All internal gaps were closed after the first round of gap closure remaining 15 contigs. These 15 contigs were ordinated in the SmR1 reference genome using nucmer, yielding 1 scaffold. After the second round of gap closure, 4 gaps remained in the genome that corresponded to repeat regions and rRNA operons and were resolved individually using blastn and FGAP. After read mapping in the assembled genome, no regions of zero coverage or structural inconsistencies were found. The genome sequence of *H. seropedicae* AU14040 was deposited in the NCBI database under accession number CP013136. The complete genome sequence of *H. seropedicae* AU14040 has 5,418,688 bp in a single circular DNA molecule with a GC% content of 63.1%. The coding sequences cover 88.3% of the genome and are distributed in 4,887 predicted protein-coding regions, 3 complete rRNA operons and 57 tRNA genes.

The initial assembly of the *Herbaspirillum* lineage 2 AU13965 genome had 38 contigs. After automatic gap closure, 12 contigs remained. These contigs were ordered in the reference genome of *H. seropedicae* SmR1, the closest in nucleotide identity, yielding 1 scaffold. The remaining gaps were closed individually as described for the AU14040 genome. At the end, we obtained two contigs corresponding to one chromosome and one plasmid. As described for the genome of *H. seropedicae* AU14040, zero coverage regions or structural inconsistencies were not found after read mapping. The genome sequence of *Herbaspirillum* lineage 2 AU13964 was deposited in the NCBI database under accession number CP034395. The closed genome of *Herbaspirillum* lineage 2 AU13965 has one chromosome of 5,350,014 bp and a GC content of 64.1%, 3 rRNA operons, 62 tRNAs and 4,720 CDSs. Its plasmid is 42,977 bp in length, containing 54 CDSs and a GC content of 62.6% and was deposited in the NCBI database under the accession number CP034394.

### Phylogenetic classification of new genomes

The original work [10] describing the bacteria used in this study classified the isolates based on 16S rRNA gene sequence analysis. To further investigate the taxonomic affiliation of the isolates AU14040 and AU13965, we calculated the average nucleotide identity (ANI) between these two genomes and the genomes of the other species of the genus *Herbaspirillum* deposited in the GenBank database (Additional file 1: Table S1). Of the 32 available genomes from the *Herbaspirillum* genus, 17 have a defined species and 15 do not. The genomes of the isolates that did not have a defined species were also included in the comparison to determine whether any of these isolates belong to the *H. seropedicae* species.

**Fig. 1:**
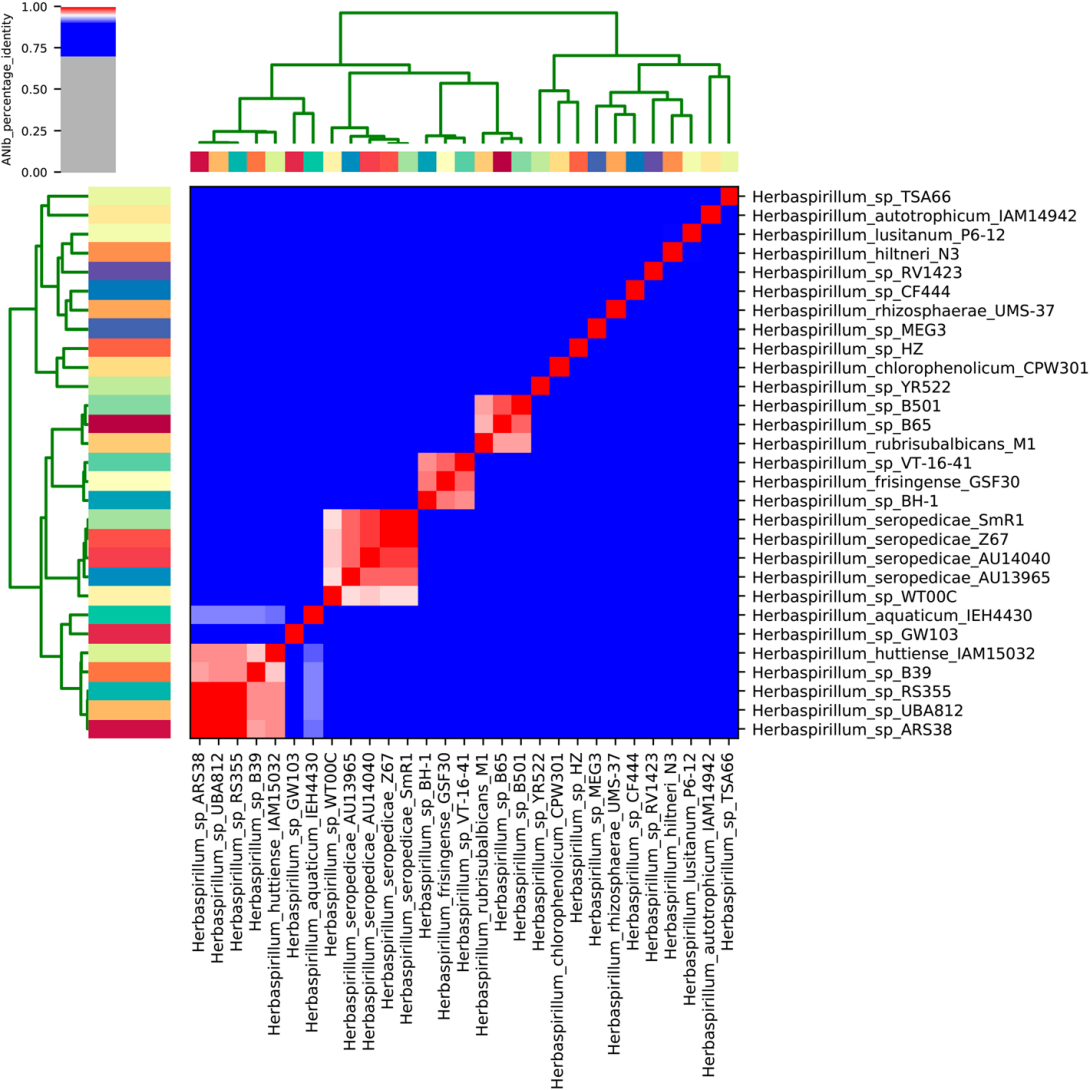
Average Nucleotide Identity (ANI) among species and strains of the genus *Herbaspirillum*. The ANI was calculated using the pyani program after blastn alignment. Only regions present in all genomes were used in the ANI calculation. The dendrogram directly reflects the degree of identity between genomes. An ANI above 95% between two genomes is an indication that they belong to the same species

The strain AU14040 was initially described as belonging to the species *H. seropedicae* and, by ANI analysis, it was possible to confirm that this isolate belongs to the species *H. seropedicae* (ANI 99%) (Fig. 1). For the isolate AU13965, on the other hand, the 16S rRNA gene sequence comparison was not sufficient to discriminate its species, which at first was classified close to the species *H. frisingense* GSF30 and *H. chlorophenolicum* CPW301 and named *Herbaspirillum* lineage 2 AU13965 [10]. However, like AU14040 strain, the ANI analysis also classified the isolate AU13965 as *H. seropedicae* (ANI 98%) (Fig. 1) and is referred to as *Herbaspirillum seropedicae* AU13965 going forward.

Among the other genomes without a defined species that were available in NCBI, the *Herbaspirillum* WT00C strain also remained in the same cluster along with *H. seropedicae* strains SmR1, Z67, AU14040 and AU13965, presenting a 96% ANI with the reference strain SmR1. These 5 genomes, 3 from environmental isolates and 2 from clinical isolates, were selected for subsequent comparative analyses. Considering strain SmR1 as the reference, clinical strains AU14040 and AU13965 showed genome reductions of 1.7 and 3%, respectively. The strain WT00C had a genome size gain of 10.2%, whereas strain Z67 was similar in size to SmR1.

### Features of the H. seropedicae AU13965 plasmid

The identification of a plasmid in the species *H. seropedicae* is unparalleled, and the plasmid in strain AU13965 is the only one described to date. Even within the genus *Herbaspirillum*, the presence of plasmids is uncommon. Looking at the GenBank database, there is only one other plasmid record for the strain *Herbaspirillum* sp. BH-1 that, according to our ANI analyses, belongs to the species *H. frisingense* (Fig. 1). Comparison of the complete AU13965 plasmid nucleotide sequence and protein amino acid sequences with the *Herbaspirillum* sp. BH-1 plasmid and proteins showed no relationship or orthologs between them. However, comparison of the AU13965 plasmid protein amino acid sequences against the GenBank database showed that 34 of the 54 proteins have orthologs (identity > 50%) in other species of the genus *Herbaspirillum*, mainly *Herbaspirillum* sp. RV1423 (Additional file 3: Table S2). In our analysis, *Herbaspirillum* sp. RV1423 was close to *Herbaspirillum hiltneri* and *Herbaspirillum lusitanum* (Fig. 1). The taxonomic distribution within the 10 best-blast hits, and not only the first hit for each protein, shows that more than 50% of the distribution is dominated by the genera *Burkholderia* (16.43%), *Herbaspirillum* (10.42%), *Chromobacterium* 7.1%), *Achromobacter* (6.43%), *Acinetobacter* (4.88%), *Neisseria* (3.10) and *Pandorea* (2.88%), all previously isolated from patients under different clinical conditions.

**Fig. 2:**
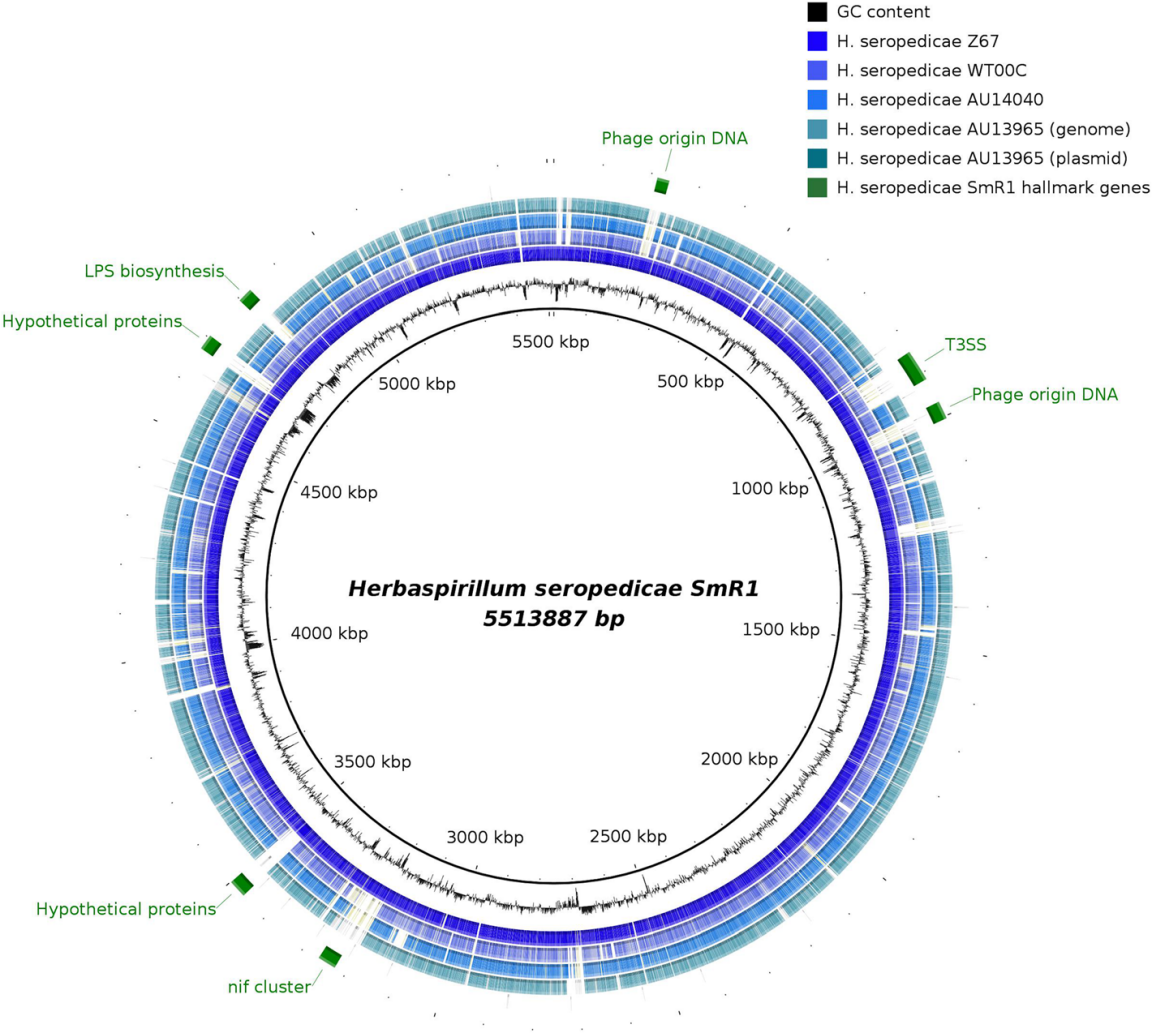
Circular comparative view of *H. seropedicae* strain genomes. Predicted proteins of strains Z67, WT00C, AU14040 and AU13965 were compared to strain SmR1, a predicted reference proteome, using blastp. The inner ring represents the SmR1 genome GC% content. The colored rings represent proteins of other strains that share at least 90% identity with SmR1 proteins. Hallmark regions of the SmR1 genome are presented in the outer ring in green

The vast majority of the proteins encoded in the plasmid were classified as hypothetical or phage-like proteins. To evaluate whether this plasmid originated from a phage genome, we performed an analysis using PHASTER [15]. According to this analysis, it was possible to verify that 26.3 kbp of the 42.9 kbp (from 15,102 to 41,457 bp) presented similarity with a phage genome (Additional file 2: Figure S1). In the genome of *H. seropedicae* SmR1, there are two large phage genome-derived inserts [6] but no correspondence between these genes and the plasmid genes were found (Fig. 2).

### Nif genes and type 3 secretion system

**Fig. 3:**
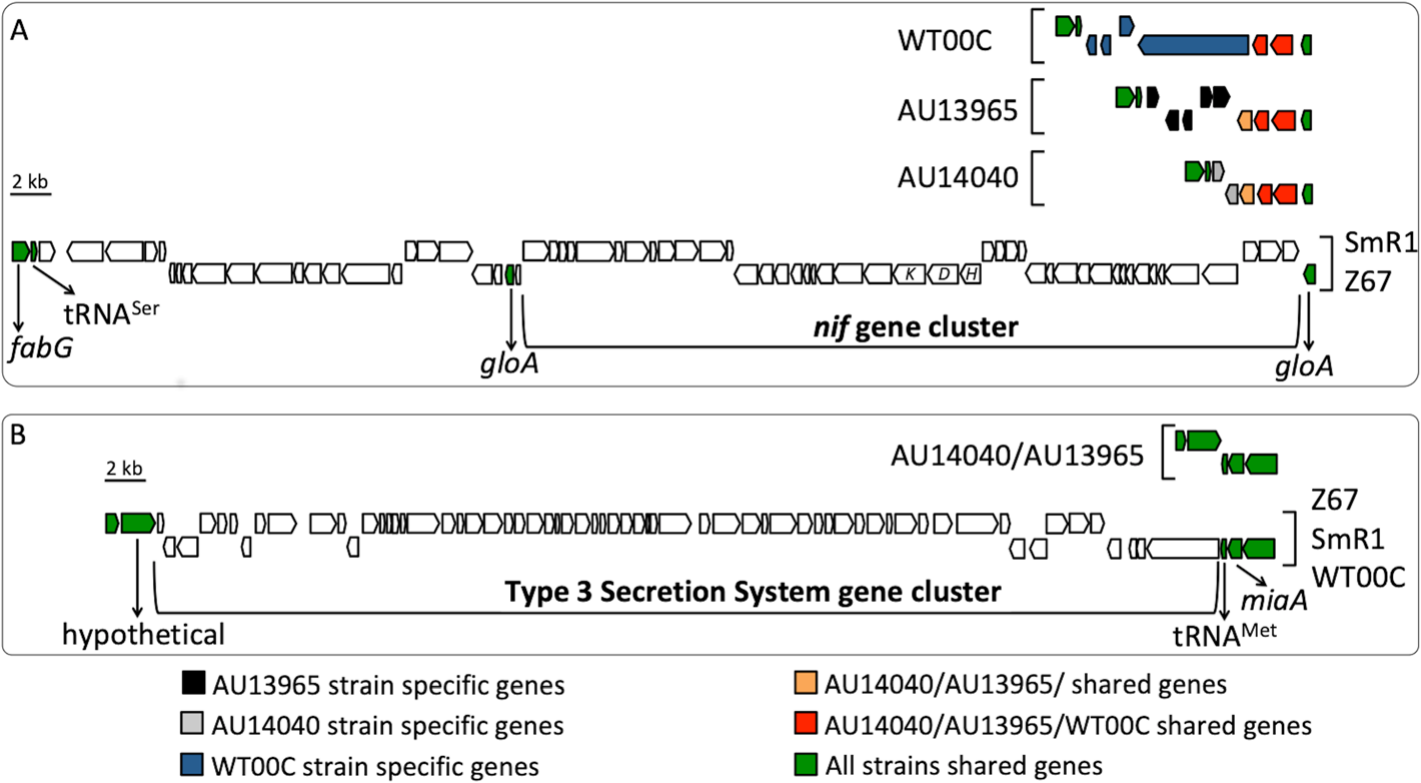
Gene clusters related to nitrogen fixation and plant association in *H. seropedicae*. The genomes of strains Z67, WT00C, AU14040 and AU13965 were aligned through blastn and visualized using the ACT tool. (**a**) The nitrogen fixation gene cluster of the SmR1 strain is located between a *gloA* gene duplication. This gene set is absent in the AU14040, AU13965 and WT00C genomes, in which only one copy of *gloA* was found. The upstream region is also absent and flanked by the *fabG* gene. (**b**) The type 3 secretion system (T3SS) is also absent in AU14040 and AU13965. In both cases, the genes flanking the absent genomic regions are conserved between strains

The *nif* gene cluster has genes related to the nitrogen fixation process, including the 3 genes that encode the structural proteins of the nitrogenase enzyme complex (NifHDK) responsible for reducing N_2_ atmospheric nitrogen to ammonia [16]. The T3SS consists of proteins that, when assembled, form a needle-like structure that is anchored in the inner membrane and crosses the outer membrane until it reaches and penetrates the plant cell, into which it injects effector proteins that modulate the host response [17]. To investigate the distribution of these gene sets within the *H. seropedicae* strains, the theoretical proteome of the SmR1 strain was used as a reference and compared to the theoretical proteomes of the other *H. seropedicae* strains using blastp (Fig. 2). With this analysis, it was possible to verify that the clinical strains did not have these two sets of genes. In the genome of the SmR1 strain, the *nif* gene cluster was located between a complete and a partial copy of the *gloA* gene, followed by a partial transposase sequence (Fig. 3a). Both genomes of the strains AU14040 and AU13965 presented only one intact copy of the *glo*A gene and no evidence of genomic insertion. A 22,700-bp region of SmR1, upstream of the *nif* cluster, was also absent in the clinical strains (Fig. 3a). In the Smr1 strain, this region was limited by a tRNA^Ser^ gene at the 3 ‘end and *glo*A at the 5’ end. The tRNA^Ser^ gene was also shared with the clinical strains; however, this region between the *glo*A and tRNA^Ser^ genes comprised 4 and 7 hypothetical proteins in strains AU14040 and AU13965, respectively. Of this set of hypothetical proteins, 3 were conserved in the clinical strains. No genes related to the *nif* cluster were found in the WT00C strain, which is in agreement with a previous analysis of the genome of this strain [18]. These results also confirm previous analyses showing that clinical strains are not able to reduce atmospheric nitrogen [11]. Considering that the *nif* gene cluster in the genus *Herbaspirillum* seems to be an early acquisition, and considering its high degree of conservation in *H. frisingense* GSF30^T^ [19] and *H. rubrisubalbicans* [20], it is probable that the *nif* genes and upstream region were lost in strains AU14040, AU13965 and, interestingly, in the environmental isolate WT00C.

The set of genes encoding the T3SS proteins appears to have been inserted into the SmR1 genome adjacent to a tRNA^Met^ (Fig. 3b). These genes were also present in the other environmental strains, Z67 and WT00C. However, this set of genes was not found in AU14040 and AU13965. Based on a comparison with the SmR1 reference genome, there were no other genes among those in the location that the system was inserted. Similar to *nif* genes, T3SS genes are also present in *H. rubrisubalbicans* [17, 20], suggesting that clinical isolates may have lost these genes.

### Production of siderophores

The strain *H. seropedicae* SmR1 has a very large gene, identified as Hsero_2343 (27,483 bp), which encodes a multimodular non-ribosomal peptide synthetase (NRPS) and produces a siderophore called serobactin [21]. However, the environmental strain SmR1 was the only strain among the analyzed genomes that has this gene intact. In strains Z67, WT00C and AU14040, this gene was divided into three genes and, in strain AU13965, into five genes. Despite the division, comparison of nucleotide sequences showed that the gene coverage of these four strains relative to the SmR1 strain was 99.5% with a minimum identity of 98%. Domain prediction analysis performed by PRISM [22] showed that, with the exception of strain AU13965, all strains presented exactly the same domains (Additional file 4: Figure S2). Apparently, the strain AU13965 shows an additional condensation domain and lost a thiolation domain. Multiple alignment of the amino acid sequences of these two regions showed that the AU13965 sequence diverges from the others. (Additional file 5: Figure S3. Although it is not possible to state whether these NRPS clusters are functional based on genomic data alone, all strains have the adenylation domains for the same amino acids and consistent with the structure determined by mass spectrometry [21].

### Global genome comparison between environmental and clinical strains

**Fig. 4:**
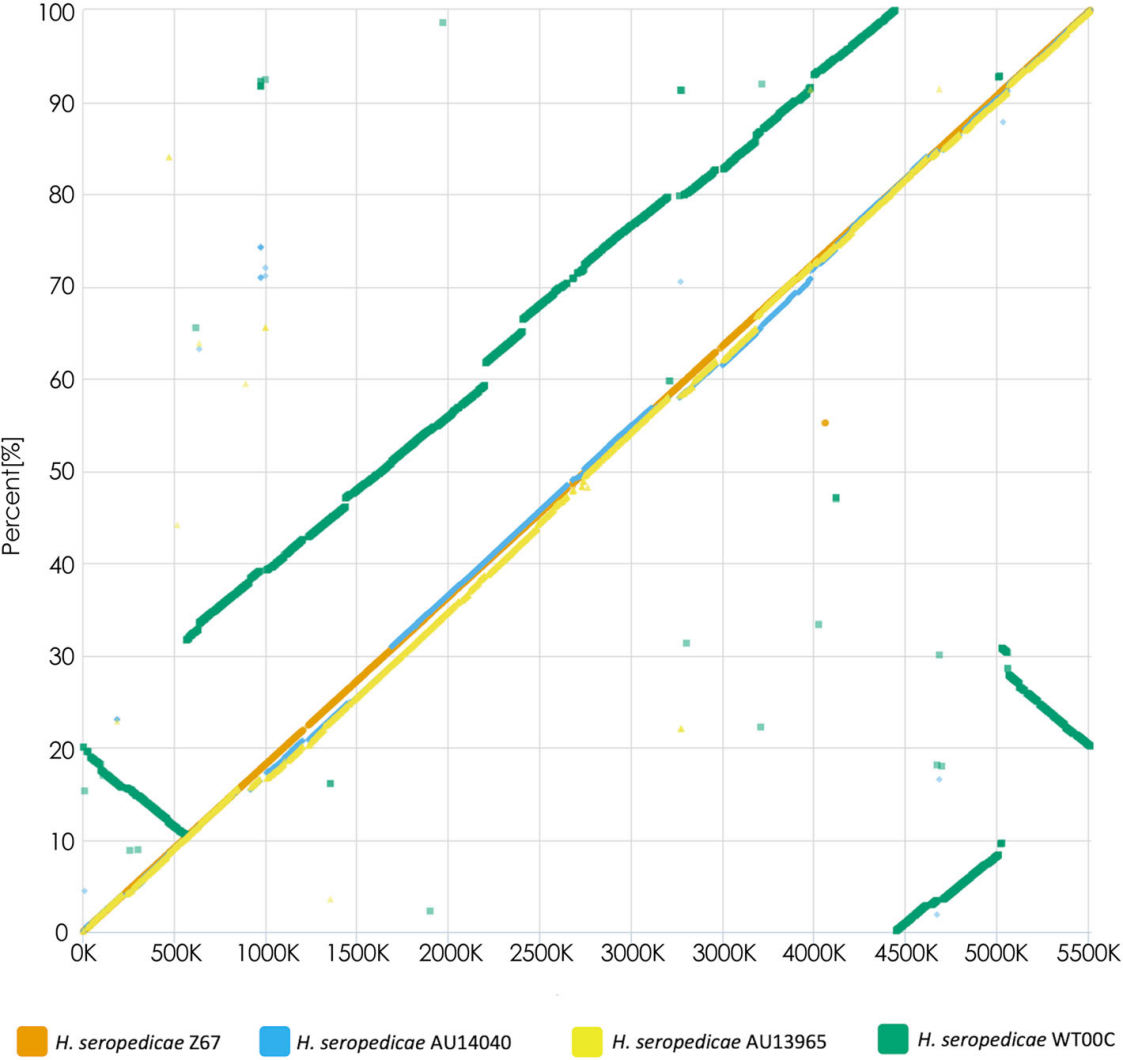
Genome sequence and structure comparison between *H. seropedicae* strains. Dotplot alignment of the six-frame translated genome sequences of strains Z67, WT00C, AU14040 and AU13965 against the SmR1 reference genome using MUMMER. The dotted line with slope equal to 1 represents an undisturbed segment of conservation between the two sequences. The closer a plot is to an imaginary line f(x) = x, the fewer macroscopic differences exist between the two sequences

Comparative analyses between the selected genomes were performed using the genome of the SmR1 strain as a reference. Synteny analysis between the genomes revealed that, among the five strains studied, SmR1, Z67, AU14040 and AU13965 show a more similar chromosomal structure. WT00C, on the other hand, presented regions of low identity, inversion and translocation, possibly reflecting the discrepancy already identified in the ANI analysis (Fig. 4).

The predicted proteome of each strain studied was functionally classified according to the KEGG groups of orthologs through the BlastKOALA tool. According to this comparison, it was possible to verify that there are no significant discrepancies between these 5 strains (Additional file 6: Figure S4). Additionally, SmR1 and Z67 had more similar numbers than WT00C, the most dissonant strain. SmR1 and Z67 had more proteins identified as environmental information and processing, including WT00C. In general, the clinical strains presented a large number of proteins classified as having a metabolic function. To verify the degree of orthology among the proteins encoded by these five strains, we performed an analysis of the core and pan genomes.

### Core and pan genome analysis

**Fig. 5:**
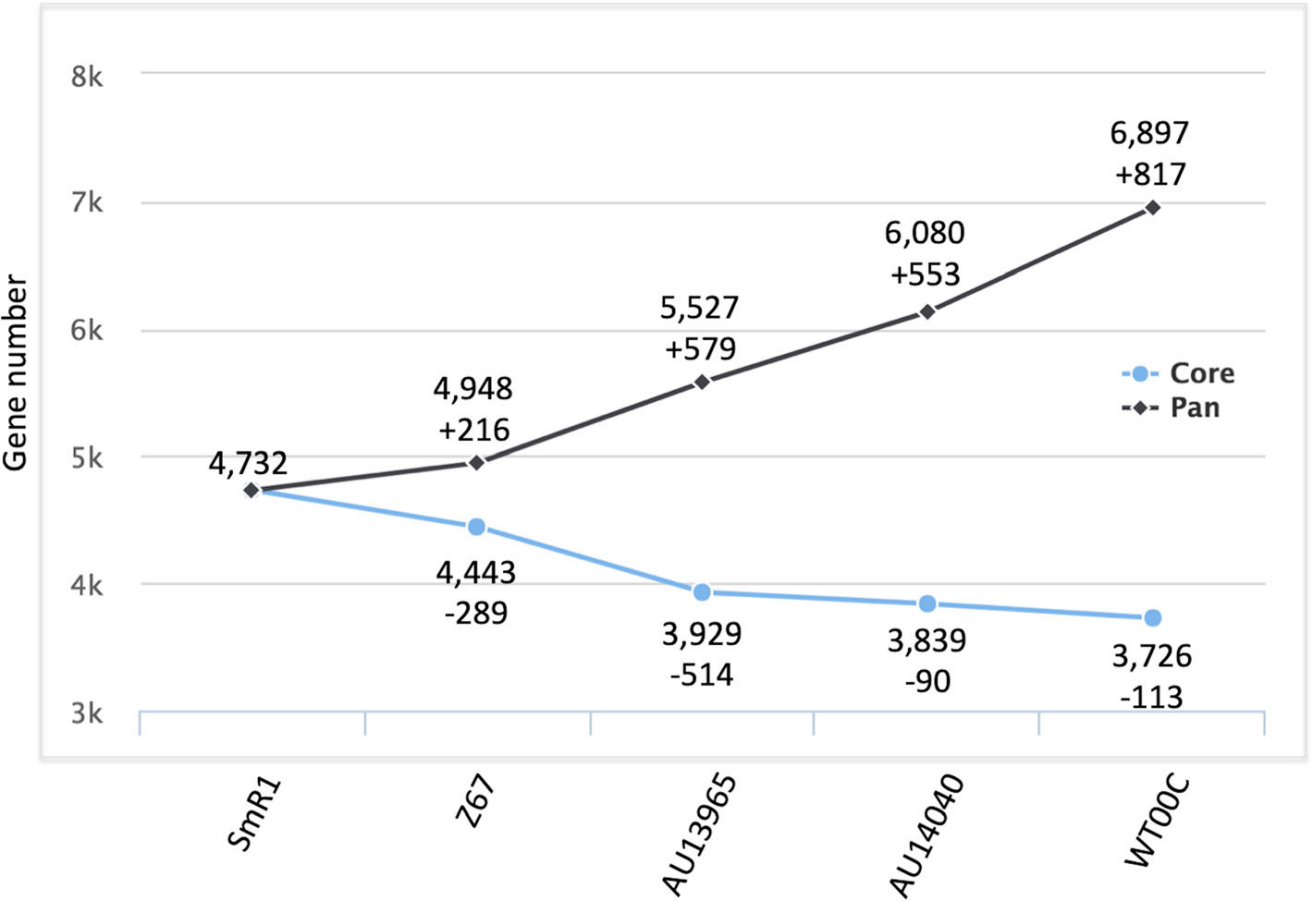
Development of the core vs pan genome. Analysis of the core and pan genomes was performed using strain SmR1 as a reference. Starting with the reference genome, the core and pan genome sizes were calculated and plotted with each new added genome based on the most frequent SRV score ratio (master SRV). This value is used to filter blastp hits within the database. Only hits with SRV values greater than or equal to the master are included

Analysis of the core and pan genomes was performed using the EDGAR2.0 software platform, using the SmR1 strain genome as a reference. The core genome development (Fig. 5) showed a decrease in the number of genes with the addition of the Z67 genome (− 289 genes), again reflecting the proximity between them. However, when the clinical strain AU13965 was included in the calculation, the core genome declined by 514 genes, indicating a greater difference between the clinical and environmental strains. Introduction of the second clinical strain, AU14040, produced no major difference (− 90 genes), suggesting that the core genome of the clinical strains is very similar. The core genome decreased by another 113 genes with the introduction of the WT00C strain, the most different among those analyzed according to the ANI and synteny results. On the other hand, gene acquisitions in the pan genome were larger than the losses of the core genome (Fig. 5). The lowest increase was observed between the SmR1 and Z67 (+ 216 genes) strains. When the clinical strains AU13965 and AU14040 and the environmental strain WT00C were added to the calculation, there was a gain of 579, 553 and 817 genes, respectively. Even between the two clinical strains, there was a large increase in the number of genes in the pan genome.

**Fig. 6:**
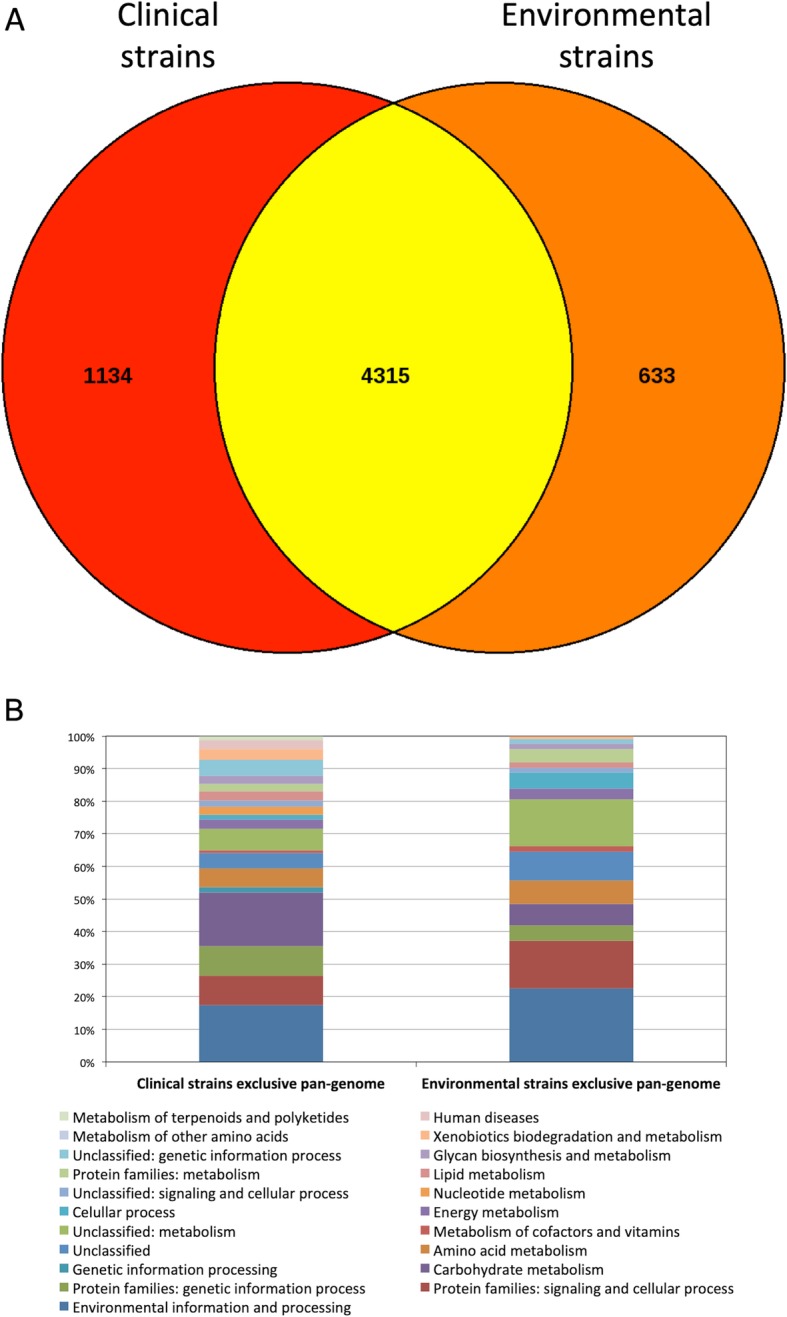
Comparison of pan genome orthologs. (**a**) Venn diagram comparing pan genome proteins between clinical and environmental strains. The exclusive sets include proteins that appeared in both or only in one genome. Proteins in the intersection set include proteins that appear in at least one clinical genome and one environmental genome. The master SRV cutoff used for classification was SRV = 33. (**b**) Functional classification of pan genome exclusive proteins of the clinical and environmental strains. Classification was made according to KEGG categories through the algorithm BlastKOALA

Considering the five strains together, the core genome had 3,727 orthologous proteins (Additional file 7: Figure S5). Again, the clinical strains AU13965 and AU14040 and the environmental strain WT00C had the highest amounts of genes (387, 482 and 813 genes, respectively) that did not have orthologs in other genomes. The SmR1 and Z67 strains presented 192 and 51 unique genes, respectively. In a more specific analysis, we coupled the gene sets of the environmental strains, SmR1 and Z67, and clinical strains, AU13965 and AU14040, to perform an orthology study. Strain WT00C was not included because it is very distant from the other 4 strains. Based on this result, it was possible to verify that the pan genome between the environmental and clinical strains contained 4,315 proteins. The pan genome exclusive of the clinical strains showed almost twice the number of proteins relative to the pan genome exclusive of the environmental strains (1,134 and 633 proteins, respectively) (Fig. 6a). Functional classification of the unique proteins from the pan genome revealed large differences between lifestyles. As in the previous comparison involving all proteins, environmental strains showed more proteins involved in the categories “Environmental information and processing”, “Signaling and cellular process” and “Metabolism”. Clinical strains showed a greater number of genes related to “Genetic information processing”, “Carbohydrate metabolism”, “Xenobiotic biodegradation and process” and “Human diseases” (Fig. 6a). Given the large number of genes involved in xenobiotic metabolism and the clinical origin of strains AU14040 and AU13695, we compared the theoretical proteomes of the strains under study against the ResFam antibiotic resistance database. The results show very similar antibiotic resistance profiles among the 5 strains, with the clinical strains presenting one or two genes more than the environmental strains (Additional file 8: Table S3). No resistance gene was found on the plasmid of strain AU13965.

### Identification of genomic islands in clinical strains

To identify potential regions in the genomes of clinical strains that may be related to adaptation to the human host, we performed a study of genomic islands. A total of 14 and 20 genomic islands were predicted in strains AU14040 and AU13965, respectively, when compared to the environmental strain SmR1. Among the 14 islands identified in AU14040 (Additional file 9: Table S4), 4 were predicted as “Strong”, 3 were classified as pathogenicity islands and 1 as a resistance island. A more detailed analysis revealed that the genes in pathogenicity islands 1 and 3 were unique to this strain, even within the genus *Herbaspirillum*. The genes present in pathogenicity island 2 and the resistance island are found in other representatives of the genus. Of the 20 islands found in AU13965, 4 were predicted as “strong”, 2 were classified as pathogenicity islands and 2 as resistance islands (Additional file 10: Table S5). The genes found in pathogenicity island 2 and resistance island 2 were unique to this strain, while the genes found in pathogenicity island 1 and resistance island 1 were identified in other bacteria of the genus. Looking specifically at pathogenicity island 3 of AU14040 and pathogenicity island 2 of AU13965, it was observed that the proteins encoded in these two islands participate in LPS biosynthesis.

Two large genes found in putative HGT regions in clinical strains may be related to pathogenicity factors. The former was identified as cyclic β-1,2-glucan synthetase (CGS) (Hsc_3506/HL2_23070) and shared 98% identity. The encoded protein had 2,853 amino acids residues and contained two in tandem glycoside hydrolase domains (GH94/ChvB/NdvB-like) plus a glycosyltransferase domain. Cyclic β glucans (CβG) are homopolysaccharides with a cyclic β 1–2 structure comprising 17–25 D-glucose residues that are exported to the periplasmic space [23]. Despite the high identity, this gene is located in distinct regions and in opposing strands in the genomes of AU14040 and AU13965. One of the functions attributed to CβG is related to bacteria-host interactions. The CGS gene is absent in SmR1 and Z67 genomes, but a highly similar protein (98% identity) was present in the genomes of WT00C strain and in the species *H. frisingense*, *H. rubrisubalbicans*, *Herbaspirillum* GW10 and *H. huttiense* sp. *putei*. Considering the high level of conservation, it is possible that the same protein produces a CβG virulence factor that is involved in both plant and mammalian colonization/interaction.

The other gene encoded a large protein with 1,452 amino acid residues in both genomes (Hsc_4347/H12_41750) and shared 97% identity. Searching the nr database using blastp, we found that there are only two species that have similar proteins: *Collimonas fungivorans* (1,561 aa, 59% similarity) and different strains of *Burkholderia gladioli* (average of 1,631 aa, 54% similarity). The translated protein had a carboxy-terminal hydrolase domain of the C19 ubiquitin peptidase (UCH) ranging from 1,056 to 1,351 aa residues. This domain is found in other proteins in the database, including various proteins of eukaryotic organisms.

### LPS biosynthesis genes are different and unique to clinical strains

**Fig. 7:**
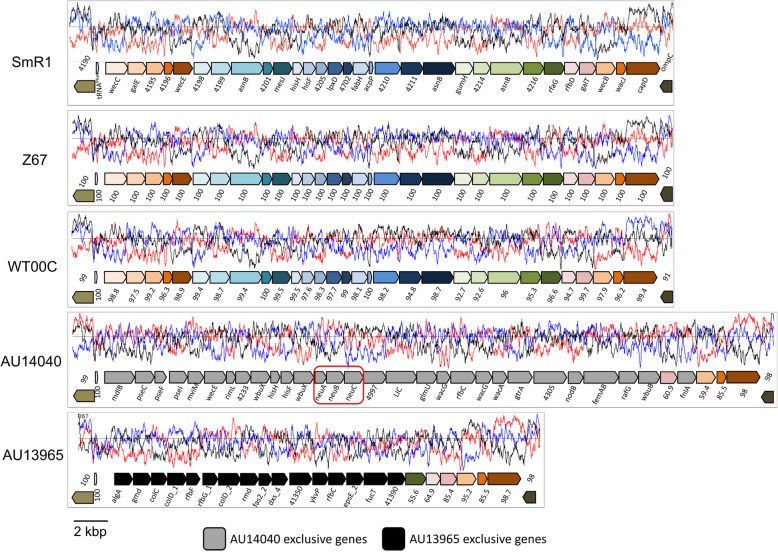
Comparison of the lipopolysaccharide (LPS) biosynthesis clusters of *H. seropedicae* strains. In all genomes, a tRNA^Phe^ gene at the 3′ end and orthologous genes at the 5′ end flank the clusters. The names in the strain SmR1 cluster correspond to the genomic annotations. Orthologous genes relative to the SmR1 strain are presented in the same colors with the level of identity below. The GC% frame plot of each region is above the genes. The black and gray arrows represent genes exclusive to strains AU1404 and AU13965, respectively. Featured in the red box are the Neu5Ac biosynthesis genes (*neuABC*) in strain AU14040

The lipopolysaccharide exposed in the outer membrane of gram-negative bacteria has different functions; among them is establishing contact with the host. In *H. seropedicae* SmR1, mutations in the gene responsible for rhamnose biosynthesis and its incorporation into LPS (*rfbB* and *rfbC*) decrease the interaction with corn roots by 100-fold [24]. In the genome of *H. seropedicae*, the region in which this gene is located is approximately 33,000 bp in size and harbors an operon of 32 genes. By performing a comparative analysis of the theoretical proteomes, it was possible to identify a gap in the genomes of the clinical strains in the region where these genes should be located (Fig. 2). A more detailed analysis of this region in the 5 genomes showed that, despite being highly conserved in the genomes of the environmental isolates SmR1, Z67 and even WT00C, the whole set of genes related to LPS biosynthesis was replaced in the clinical isolates (Fig. 7). However, unlike the conservation seen in environmental isolates, there was no conservation among the vast majority of genes in that region among clinical isolates. The exceptions were the upstream tRNA^Ser^ and the last 3 genes of the operon, which were conserved among the 5 genomes and may constitute recombination sites. Interestingly, in all genomes, the region containing genes related to LPS biosynthesis presented GC content below the genome average, indicating that this region is derived from lateral transfer and apparently undergoes easy recombination.

A peculiarity found in the AU14040 strain LPS cluster was the presence of genes related to the biosynthesis of N-acetyl neuraminic acid (Neu5Ac). Neu5Ac is an important constituent of the mammalian cell surface but is not synthesized by most bacterial cells. Pathogenic species are exceptions, covering their cell surface with Neu5Ac as a way of mimicking the surface of the host cell to avoid the immune system [25]. The biosynthesis of Neu5Ac in bacteria is performed by three enzymes: NeuA, an epimerase; NeuB, which catalyzes the condensation of phosphoenol pyruvate and N-acetyl mannose forming the Neu5Ac itself; and NeuC, which activates Neu5Ac through CMP binding, producing Neu5Ac-CMP [25]. In the genome of strain AU14040, the ORFs Hsc_4294, Hsc_4295 and Hsc_4296 encoded these enzymes, respectively. Downstream of the *neuC* gene was a gene encoding a surface carbohydrate biosynthesis protein (Hsc_4297), a transferase from the family Lic12162 (Hsc_4298) and a family 1 glycosyltransferase (Hsc_4299), which could be involved in the transfer of Neu5Ac to the LPS structure. It was also possible to identify genes encoding the pseudaminic acid biosynthesis enzymes (*pseCFI*, ORs Hsc_4283–4284-4285) and N-acetylfucosamine, suggesting that it may be part of the LPS structure. As previously described, the genes found in this island were exclusive to this strain.

Another way in which pathogenic bacteria acquire Neu5Ac, in addition to biosynthesis, is through the transport of this molecule through the membrane by TRAP (tripartite ATP-independent periplasmic transport)-type carriers formed by a Neu5Ac binding protein (SiaP), two transmembrane proteins (SiaQ) and a conserved protein with 12 transmembrane domains (SiaM) [26]. Unlike the *neuABC* genes, all *H. seropedicae* genomes included in this study presented two distinct copies of the *siaPQM* operon (Additional file 11: Table S6), while the SiaQM proteins fused into a single 16 transmembrane domain protein (SiaT). This configuration was also found in *Haemophilus influenza*, in which the relationship between the *siaPQM* operon and pathogenicity was first described [27].

## Discussion

SmR1, Z67, WT00C, AU13965 and AU14040 are strains of the species *Herbaspirillum seropedicae*, although they were isolated from remarkably different environments. SmR1 and Z67 are environmental strains capable of establishing an endophytic association with grass plants and fixing nitrogen. AU14040 and AU13965, on the other hand, are two strains isolated from the sputum of a patient with cystic fibrosis. Sequencing, assembly and analysis of the AU14040 and AU13965 genomes showed that clinical strains lack two gene clusters that are considered hallmarks of the *Herbaspirillum*-plant interaction: the *nif* cluster and T3SS. A similar case was reported in a genomic analysis involving more than 300 genomes of bacteria from the genus *Klebsiella* [13]. The *nif* gene cluster was found in all genomes analyzed from phylogenetic group III (*Klebsiella variicola*), which is described as an environmental nitrogen-fixing group. However, the *nif* gene cluster was found in only one genome of phylogroup I (*Klebsiella pneumoniae*), described as capable of infecting humans and other mammals. Despite the different niches, all *Klebsiella* genomes analyzed have at least 96% average nucleotide identity [13]. Strain AU13965, in turn, contained a plasmid, which is the first described and analyzed within the genus *Herbaspirillum*, showing potential for the transfer of genetic material.

Some prokaryotes are capable of producing low-molecular-weight iron affinity molecules called siderophores. These small molecules are released into the extracellular medium and, after binding to iron ions, are internalized again through membrane-specific transporters [28]. The SmR1 strain has a very large gene identified as a NRPS. Mutations in this gene produce a strain incapable of assimilating iron [29]. In the clinical strains this gene is fragmented and, despite having the adenylation domain for the same amino acids, strain AU13965 shows an additional condensation domain and lacks a thiolation domain. The production of siderophores and the uptake of iron are considered important virulence factors in pathogens that infect humans, such as *Klebsiella pneumoniae* [30], *Yersinia pestis* [31] and *Neisseria gonorrhoeae* [32]. Furthermore, in *K. pneumoniae*, the presence and number of genes related to siderophore production is directly associated with invasive strains relative to noninvasive strains. The association between iron uptake and virulence is related to the low bioavailability of this ion in the host, and infectious agents need to optimize their capture and transport processes [33].

Lifestyle and host changes are drastic events followed by loss and acquisition of genes. The search for regions of HGT and genomic islands identified three genomic regions that may be involved in this process. The CβG produced by CGS is related to bacteria-host interaction, whether symbiotic or pathogenic. The CβGs produced by *Xanthomonas campestris* sp. *campestris* during infection of *Nicotiana benthamiana* and *Arabidopsis thaliana* are transported to the plant and act to suppress the plant immune system by reducing the expression of pathogen-related (PR-1) proteins [34]. The CβG produced by *Brucella abortus* during mammalian cell infection allows bacterial replication inside the host cell and prevents lysosome fusion [35]. In both cases, CβG is an essential factor for virulence, and mutants for CβG production are less infective and more susceptible to clearance. The other CDS found in a potential HGT region was the ubiquitin carboxi-terminal hydrolase (UCH). The ubiquitin-autophagy pathway may act as an innate immune response system to ubiquitin-labeled intracellular pathogens that escape lysosomal fusion and redirect them to degradation [36, 37]. As the CGS, the UCH could assist the bacterium to evade immune system. Surprisingly, the UCH has similarity with proteins of eukaryotic origin. The identification of eukaryotic-like proteins in pathogenic bacteria has been described in *Legionella pneumophila*, the causative agent of legionnaires’ disease. Genome analyses found 30 genes encoding eukaryotic-like proteins and 32 other eukaryotic-like domains that modulate the host cell for the benefit of the pathogen [38]. Finally, a striking difference was found in the region where the genes related to LPS biosynthesis are located. One function of the LPS is mediate the bacterium-host interaction. In our analysis, we verified that the LPS gene cluster in clinical strains was substituted when compared to environmental ones. Even among clinical strains, there is no similarity between the genes encoded within the LPS cluster. An interesting feature found in the LPS gene cluster of strain AU14040 was de presence of the *neuABC* operon and a LIC family transferase. The NeuABC proteins are responsible biosynthesis and activation of the Neu5Ac, whereas the LIC transferase add it to LPS [25]. Pathogenic bacteria use the Neu5Ac-linked LPS as a manner of mimicry the human host cells surface and evade immune system [39]. Similar alteration of the LPS gene cluster was described in *Burkholderia* isolates [40]. In this case, nonpathogenic *Burkholderia thailandensis* acquired a cluster of biosynthesis genes from the capsular polysaccharide of *Burkholderia pseudomallei* through HGT. This cluster spread among this line, forming a subgroup of *B. thailandensis* with characteristics common to *B. pseudomallei* and distinct from ancestral *B. thailandensis*, representing an improvement in intracellular macrophage survival. In another study, Feng et al. showed that the inactivation of the *neuB* gene in *Streptococcus suis* leads to a decrease in Neu5Ac production and increased phagocytosis by macrophages [41].

## Conclusions

Clinical isolates of *Herbaspirillum*, mainly from immunocompromised patients, have been considered opportunistic pathogens to date. However, these new genomic data findings suggest that the lifestyle transition from environment to opportunist led to the loss and acquisition of specific genes to allow adaptations to colonize and survive in new environments. Of all differences, the substitution of the of gene cluster related to LPS biosynthesis seems to be the starting point for the colonization of human hosts.

## Methods

### Purification and sequencing of genomic DNA

The bacterial strains used in this study were obtained from the *Burkholderia cepacia* Research Laboratory and Repository (BcRLR, University of Michigan, Ann Arbor) and kindly provided by Dr. John LiPuma. *H. seropedicae* AU14040 and *Herbaspirillum* lineage 2 cells were cultured in NFbHP liquid medium at 37 **°**C, and chromosomal DNA was purified using the phenol-chloroform method [6]. The genome of *H. seropedicae* AU14040 was sequenced on the MiSeq (Illumina) platform using the 2X250 bp paired-end configuration, the SOLiD 4 platform (Life Technologies) using the 2X50 bp mate-pair configuration and the PGM Ion Torrent platform (Life Technologies) using the single-end 200 bp configuration. The genome of the *Herbaspirillum* lineage 2 was sequenced on the Illumina MiSeq platform using the 2X300 bp paired-end configuration and the Ion Proton platform using the 200 bp single-end configuration. For the construction of sequencing libraries, we used Nextera sample prep kits (Illumina) for the MiSeq platform and the Ion Xpress Plus Fragment Library Kit (ThermoFisher Scientific) for the Ion Proton platform.

### Genome assembly, finalization and annotation

The genome assembly of each isolate was performed individually using the separate reads from the different sequencing platforms and in a combined manner using the reads of all sequencing platforms in the same assembly. We used the Newbler v2.9 assembler (454 Life Sciences, Brandford, CT), SPAdes v3.10.0 [42] and the De novo CLC assembler v10 (CLC bio, Qiagen). Evaluation of the assemblies was performed by using the QUAST (Quality Assessment Tool for Genome Assemblies) program [43] to choose the best result, which was used as the main assembly for the next steps. Gap filling for this assembly was performed with the GFinisher [44] and FGAP [45] programs using other assemblies as a dataset. The gaps remaining after this step were manually closed by looking for regions of similarity at the ends of the contigs using the blastn algorithm [46] and nucmer tool of the MUMMER package [47]. Validation of the assembly was performed by mapping the reads in the final assembly, using the CLC bio mapping tool, to identify regions of inconsistencies in the genome structure. The genomes were annotated using Prokka [48].

### Genomic comparisons

Comparison of the nucleotide sequences of the genomes was performed by calculating the average nucleotide identity (ANI) using the pyani algorithm [49]. The ANI value shows strong correlation with 16S rRNA identity and DNA-DNA hybridization for bacterial species identification and taxonomy. An ANI value equal to or greater than 95% between two genomes is considered indicative that they belong to the same species [50]. The similarities and divergences between the genomes of the clinical and environmental strains were investigated through a genome-genome alignment performed with the blastn [46] and blastp [51] algorithms and visualized through the Artemis Comparison Tool - ACT [52] and Blast Ring Image Generator (BRIG) [53].

### Orthology analysis

Identification of orthologous genes and determination of the core and pan-genome was performed using the EDGAR software platform [54]. The core genome of a given taxonomic group is defined as the set of genes present in all genomes. On the other hand, the pan genome is defined as the total set of genes that represent a particular taxonomic group. The clustering method used by EDGAR is based on bi-directional blast and a calculated score ratio value (SRV). The SRV method uses a normalization approach by relating all bit scores of a protein to the maximum bit score that can be achieved by this protein sequence [54].

### Identification of genomic islands

Potential regions of horizontal gene transfer (HGT) and genomic islands were identified through the algorithms Alien Hunter [55] and GIPSy [56], respectively. The Alien Hunter program use the Interpolated Variable Order Motifs (IVOMs) approach to predict putative regions of HGT based on the sequence composition without a reference genome. The GIPSY program, on the other hand, predicts genomic islands by comparing the genome of interest with a reference genome. Only the regions absent in the reference genome are considered, as well as the % GC, the use of codons and the presence of moving elements.

### Functional classification

The BlastKOALA algorithm [57] was used for gene functional classification according to the KEGG groups of orthologs. The HMMer [58] v3.1b2 algorithm was used to search for genes that confer resistance to antibiotics in the HMM profile of a curated bank of antibiotic resistance genes, ResFams (Core v1.2) [59]. Non-ribosomal peptide synthetase domain identification was performed using PRISM [22].

### Nucleotide accession number

The sequenced and assembled genomes of *H. seropedicae* strains AU14040 and AU13965 were deposited in the Genbank database under accession numbers CP013136 and CP034394, respectively.

## Additional files

Additional file 1: **Table S1.** Genomes of species and strains of the genus *Herbaspirillum* used in the ANI calculation. (DOCX 38 kb)

Additional file 2: **Figure S1.** Map of the strain AU13965 plasmid. Approximately 26.3 kbps (Region 1) were identified as phage-derived sequences. Genes encoding putative phage proteins were also identified. (DOCX 95 kb)

Additional file 3: **Table S2.** Best-blast hits of AU13965 plasmid-encoded proteins. (DOCX 93 kb)

Additional file 4: **Figure S2.** Identification of the non-ribosomal peptide synthetase (NRPS) cluster in *H. seropedicae* strains. Sequences of the NRPS cluster proteins encoded in each genome were identified based on the sequences of SmR1 proteins. The retrieved sequences were analyzed by PRISM for annotation and identification of functional domains. Adenylation domains are: OHBu: 3-hydroxybutanoic acid; Asp: Aspartate; Ser: Serine; Thr: Threonine; OHOm: N5-hydroxyornithine. Other biosynthetic domains are: TE: Thioesterase; PPTase: Phosphopantetheinyltransferase; T: Thiolation C: condensation; E: Epimerization. (DOCX 377 kb)

Additional file 5: **Figure S3.** Multiple sequence alignment of NRPS. The amino acid sequences of the NRPS genes were aligned using Clustal Omega. (A) Additional condensation site region of AU13965. (B) Region of the thiolation site absent in strain AU13965. (DOCX 207 kb)

Additional file 6: **Figure S4.** Functional classification of *Herbaspirillum* strain genomes. Functional classification of proteins from clinical and environmental strains was made according to the KEGG categories through the algorithm BlastKOALA. The number of CDSs in each category was normalized by the total number of CDSs in each strain. (DOCX 69 kb)

Additional file 7: **Figure S5.** Identification of orthologous proteins in *H. seropedicae* strains. Venn diagram of orthologous proteins between clinical and environmental strains of *Herbaspirillum seropedicae*. The theoretical proteomes of all strains were compared to each other using blastp. From the blastp results, SRV was calculated. The master SRV cutoff used for clustering was 33. The analysis was performed with the EDGAR software platform. (DOCX 194 kb)

Additional file 8: **Table S3.** Comparison of the theoretical proteome of *H. seropedicae* strains with the ResFam antibiotic resistance gene database. (DOCX 49 kb)

Additional file 9: **Table S4.** Genomic islands of AU14040 strains predicted as “strong”. (DOCX 83 kb)

Additional file 10: **Table S5.** Genomic islands of AU13965 strains predicted as “strong”. (DOCX 73 kb)

Additional file 11: **Table S6.** Neu5Ac metabolism in *Herbaspirillum seropedicae*. (DOCX 55 kb)

## Data Availability

All genomes used in this work, including those from the clinical strains, are publicly available in the GenBank database.

## Abbreviations

ANI: Average Nucleotide Identity
CbG: Cyclic β glucan
CDS: Coding Sequence
CGS: Cyclic β-1,2-glucan synthetase
HGT: Horizontal Gene Transfer
LPS: Lipopolysaccharide
N_2_: nitrogen
Neu5Ac: N-acetylneuraminic acid
NH^4+^: ammonium
*nif*: nitrogen fixation gene cluster
NRPS: Non-ribosomal peptide synthetase
rRNA: ribosomal RNA
SRV: Score Ratio Value
T3SS: Type 3 secretion system
tRNA: transfer RNA
UCH: Ubiquitin Carboxil hydrolase
