# Supplementary material for: Genome comparison between clinical and environmental strains of Herbaspirillum seropedicae reveals a potential new emerging bacterium adapted to human hosts
Source: BMC Genomics. 2019 Aug 2;20:630. doi: 10.1186/s12864-019-5982-9 (PMC6679464; doi:10.1186/s12864-019-5982-9)
Supplement: Supplementary file 1 — Table S1. Genomes of species and strains of the genus Herbaspirillum used in the ANI calculation. (DOCX 38 kb) [file 12864_2019_5982_MOESM1_ESM.docx]

**Additional file 1:**

**Table S1: Genomes of species and strains of the genus *Herbaspirillum* used in the ANI calculation.**

| Organism | Strain | Acc. Number | Organism source | Reference | Country of isolation | |
| --- | --- | --- | --- | --- | --- | --- |
| *H. seropedicae* | SmR1 | CP002039.1 | Grass endophyte | (1) | Brazil |  |
| *H. seropedicae* | Z67^T^ | CP011930 | Grass endophyte | (2) | Brazil |  |
| *H. rubrisubalbicans* | M1 | CP013737.1 | Sugarcane endophyte | (3) | Brazil |  |
| *H. frisingense* | GSF30^T^ | AEEC02 | C4-fibre plants | (4) | Germany |  |
| *H. chlorophenolicum* | CPW301^T^ | LFLT0.1 | Soil sediments | (5) | South Korea |  |
| *H. hiltneri* | N3 | CP011409.1 | Rice surface | (6) | Germany |  |
| *H. huttiense* sup. *putei* | IAM15032^T^ | ANJR0.1 | Well water | (7) | Japan |  |
| *H. lusitanum* | P6-12^T^ | AJHH0.1 | Beans nodule | (8) | Portugal |  |
| *H. rhizosphaerae* | UMS-37 | LFLU0.1 | Garlic rhizosphere | (9) | South Korea |  |
| *H. aquaticum* | IEH4430^T^ | NJGV0.1 | Deionized water | (7) | United States |  |
| *H. autotrophicum* | IAM14942 | LFLS0.1 | Eutrophic lake | (10) | Switzerland |  |
| *Herbaspirillum* sp. | YR522 | AKJA0.1 | Populus endosphere | (11) | United States |  |
| *Herbaspirillum* sp. | GW103 | AJVC0.1 | Phragmites rhizosphere | (12) | Korea |  |
| *Herbaspirillum* sp. | CF444 | AKJW0.1 | Populus endosphere | (11) | United States |  |
| *Herbaspirillum* sp. | B39 | BADF0.1 | Rice shoot | - | Japan |  |
| *Herbaspirillum* sp. | B501 | BADJ0.1 | Rice shoot | - | Japan |  |
| *Herbaspirillum* sp. | RV1423 | CBXX0.1 | hydrocarbon-contaminated water | (13) | Czech Republic |  |
| *Herbaspirillum sp.* | UBA812 | DBIC01.1 | Marine metagenome | (14) | - |  |
| *Herbaspirillum sp.* | RS355 | PBPP0.1 | Marine metagenome | (15) | - |  |
| *Herbaspirillum sp.* | ARS38 | NZAE0.1 | Marine metagenome | (15) | - |  |
| *Herbaspirillum sp.* | MEG3 | CP022736 | Soil | (16) | South Korea |  |
| *Herbaspirillum sp.* | HZ10 | NJGU01.1 | Robinia pseudoacacia root nodule | (17) | China |  |
| *Herbaspirillum sp.* | WT00C | MIJG0.1 | Camellia sinensis L. tissues | (18) | China |  |
| *Herbaspirillum sp.* | VT1641 | MUXB0.1 | Urinary tract | (19) | United States |  |
| *Herbaspirillum sp.* | TSA66 | JWJG0.1 | Rice paddy soils | (20) | Japan |  |
| *Herbaspirillum* sp. | B65 | BADI0.1 | Rice shoot | - | Japan |  |
| *Herbaspirillum* sp. | BH-1 | PKOI0.1 | Biuret enriched soil | (21) | United States |  |

**Additional file references**

1. Pedrosa FO, Monteiro RA, Wassem R, Cruz LM, Ayub RA, Colauto NB, et al. Genome of Herbaspirillum seropedicae Strain SmR1, a Specialized Diazotrophic Endophyte of Tropical Grasses. PLoS Genet. 2011;12;7(5):e1002064.

2. Baldani JI, Baldani VLD, Seldin L, Dobereiner J. Characterization of Herbaspirillurn seropedicae gen. nov. sp. nov. a Root- Associated Nitrogen-Fixing Bacterium. Int J Syst Bacteriol. 1986;36(1):8.

3. Baldani JI, Pot B, Kirchhof G, Falsen E, Baldani VL, Olivares FL, et al. Emended description of Herbaspirillum; inclusion of [Pseudomonas] rubrisubalbicans, a milk plant pathogen, as Herbaspirillum rubrisubalbicans comb. nov.; and classification of a group of clinical isolates (EF group 1) as Herbaspirillum species 3. Int J Syst Bacteriol. 1996;46(3):802–10.

4. Kirchhof G, Eckert B, Stoffels M, Baldani JI, Reis VM, Hartmann A. Herbaspirillum frisingense sp. nov., a new nitrogen-fixing bacterial species that occurs in C4-fibre plants. Int J Syst Evol Microbiol. 2001;51(Pt 1):157–68.

5. Im WT, Bae HS, Yokota A, Lee ST. Herbaspirillum chlorophenolicum sp. nov., a 4-chlorophenol-degrading bacterium. Int J Syst Evol Microbiol. 2004;54(Pt 3):851–5.

6. Rothballer M, Schmid M, Klein I, Gattinger A, Grundmann S, Hartmann A. Herbaspirillum hiltneri sp. nov., isolated from surface-sterilized wheat roots. Int J Syst Evol Microbiol. 2006;56(6):1341–8.

7. Dobritsa AP, Reddy MCS, Samadpour M. Reclassification of Herbaspirillum putei as a later heterotypic synonym of Herbaspirillum huttiense, with the description of H. huttiense subsp. huttiense subsp. nov. and H. huttiense subsp. putei subsp. nov., comb. nov., and description of Herbaspirillum. Int J Syst Evol Microbiol. 2010;60(6):1418–26.

8. Valverde A, Velázquez E, Gutiérrez C, Cervantes E, Ventosa A, Igual JM. Herbaspirillum lusitanum sp. nov., a novel nitrogen-fixing bacterium associated with root nodules of Phaseolus vulgaris. Int J Syst Evol Microbiol. 2003;53(Pt 6):1979–83.

9. Jung SY, Lee MH, Oh TK, Yoon JH. Herbaspirillum rhizosphaerae sp. nov., isolated from rhizosphere soil of Allium victorialis var. platyphyllum. Int J Syst Evol Microbiol. 2007;57(10):2284–8.

10. Ding L, Yokota A. Proposals of Curvibacter gracilis gen. nov., sp. nov. and Herbaspirillum putei sp. nov. for bacterial strains isolated from well water and reclassification of [Pseudomonas] huttiensis, [Pseudomonas] lanceolata, [Aquaspirillum] delicatum and [Aquaspirillum. Int J Syst Evol Microbiol. 2004;54(Pt 6):2223–30.

11. Brown SD, Utturkar SM, Klingeman DM, Johnson CM, Martin SL, Land ML, et al. Twenty-one genome sequences from pseudomonas species and 19 genome sequences from diverse bacteria isolated from the rhizosphere and endosphere of Populus deltoides. Vol. 194, Journal of Bacteriology. 2012. p. 5991–3.

12. Lee GW, Lee KJ, Chae JC. Genome sequence of Herbaspirillum sp. strain GW103, a plant growth-promoting bacterium. J Bacteriol. 2012;194(15):4150.

13. Jauregui R, Rodelas B, Geffers R, Boon N, Pieper DH, Vilchez-Vargas R. Draft Genome Sequence of the Naphthalene Degrader Herbaspirillum sp. Strain RV1423. Genome Announc. 2014;2(2).

14. Parks DH, Rinke C, Chuvochina M, Chaumeil PA, Woodcroft BJ, Evans PN, et al. Recovery of nearly 8,000 metagenome-assembled genomes substantially expands the tree of life. Nat Microbiol. 2017;2(11):1533–42.

15. Tully BJ, Graham ED, Heidelberg JF. The reconstruction of 2,631 draft metagenome-assembled genomes from the global oceans. Sci Data. 2018;5.

16. Kim YE, Do KT, Unno T, Park SJ. Complete genome sequence of Herbaspirillum sp. meg3 isolated from soil. Korean J Microbiol. 2017;53(4):326–8.

17. Fan MC, Guo YQ, Zhang LP, Zhu YM, Chen WM, Lin YB, et al. Herbaspirillum robiniae sp. nov., isolated from root nodules of Robinia pseudoacacia in a lead-zinc mine. Int J Syst Evol Microbiol. 2018;68(4):1300–6.

18. Cheng W, Zhan G, Liu W, Zhu R, Yu X, Li Y, et al. Draft Genome Sequence of Endophytic Herbaspirillum sp. Strain WT00C, a Tea Plant Growth-Promoting Bacterium. Genome Announc. 2017;5(11):e01719-16.

19. Tetz V, Tetz G. Draft genome sequence of the uropathogenic Herbaspirillum frisingense strain ureolyticus VT-16-41. Genome Announc. 2017;5(17).

20. Ishii S, Ashida N, Otsuka S, Senoo K. Isolation of oligotrophic denitrifiers carrying previously uncharacterized functional gene sequences. Appl Environ Microbiol. 2011;77(1):338–42.

21. Robinson SL, Badalamenti JP, Dodge AG, Tassoulas LJ, Wackett LP. Microbial biodegradation of biuret: defining biuret hydrolases within the isochorismatase superfamily. Environ Microbiol. 2018;20(6):2099–111.
