# Supplementary material for: Genome comparison between clinical and environmental strains of Herbaspirillum seropedicae reveals a potential new emerging bacterium adapted to human hosts
Source: BMC Genomics. 2019 Aug 2;20:630. doi: 10.1186/s12864-019-5982-9 (PMC6679464; doi:10.1186/s12864-019-5982-9)
Supplement: Supplementary file 2 — Figure S1. Map of the strain AU13965 plasmid. Approximately 26.3 kbps (Region 1) were identified as phage-derived sequences. Genes encoding putative phage proteins were also identified. (DOCX 95 kb) [file 12864_2019_5982_MOESM2_ESM.docx]

**Additional file 2:**

**Figure S1: Map of the strain AU13965 plasmid.** Approximately 26.3 kbps (Region 1) were identified as phage-derived sequences. Genes encoding putative phage proteins were also identified.
