# Supplementary material for: Genome comparison between clinical and environmental strains of Herbaspirillum seropedicae reveals a potential new emerging bacterium adapted to human hosts
Source: BMC Genomics. 2019 Aug 2;20:630. doi: 10.1186/s12864-019-5982-9 (PMC6679464; doi:10.1186/s12864-019-5982-9)
Supplement: Supplementary file 3 — Table S2. Best-blast hits of AU13965 plasmid-encoded proteins. (DOCX 93 kb) [file 12864_2019_5982_MOESM3_ESM.docx]

**Additional file 3:**

**Table S2: Best-blast hits of AU13965 plasmid-encoded proteins.**
