# Supplementary material for: Genome comparison between clinical and environmental strains of Herbaspirillum seropedicae reveals a potential new emerging bacterium adapted to human hosts
Source: BMC Genomics. 2019 Aug 2;20:630. doi: 10.1186/s12864-019-5982-9 (PMC6679464; doi:10.1186/s12864-019-5982-9)
Supplement: Supplementary file 4 — Figure S2. Identification of the non-ribosomal peptide synthetase (NRPS) cluster in H. seropedicae strains. Sequences of the NRPS cluster proteins encoded in each genome were identified based on the sequences of SmR1 proteins. The retrieved sequences were analyzed by PRISM for annotation and identification of functional domains. Adenylation domains are: OHBu: 3-hydroxybutanoic acid; Asp: Aspartate; Ser: Serine; Thr: Threonine; OHOm: N5-hydroxyornithine. Other biosynthetic domains are: TE: Thioesterase; PPTase: Phosphopantetheinyltransferase; T: Thiolation C: condensation; E: Epimerization. (DOCX 377 kb) [file 12864_2019_5982_MOESM4_ESM.docx]

**Additional file 4:**

**Figure S2: Identification of the non-ribosomal peptide synthetase (NRPS) cluster in *H. seropedicae* strains.** Sequences of the NRPS cluster proteins encoded in each genome were identified based on the sequences of SmR1 proteins. The retrieved sequences were analyzed by PRISM for annotation and identification of functional domains. Adenylation domains are: OHBu: 3-hydroxybutanoic acid; Asp: Aspartate; Ser: Serine; Thr: Threonine; OHOm: N5-hydroxyornithine. Other biosynthetic domains are: TE: Thioesterase; PPTase: Phosphopantetheinyltransferase; T: Thiolation C: condensation; E: Epimerization.
