# Supplementary material for: Genome comparison between clinical and environmental strains of Herbaspirillum seropedicae reveals a potential new emerging bacterium adapted to human hosts
Source: BMC Genomics. 2019 Aug 2;20:630. doi: 10.1186/s12864-019-5982-9 (PMC6679464; doi:10.1186/s12864-019-5982-9)
Supplement: Supplementary file 5 — Figure S3. Multiple sequence alignment of NRPS. The amino acid sequences of the NRPS genes were aligned using Clustal Omega. (A) Additional condensation site region of AU13965. (B) Region of the thiolation site absent in strain AU13965. (DOCX 207 kb) [file 12864_2019_5982_MOESM5_ESM.docx]

**Additional file 5:**

**Figure S3: Multiple sequence alignment of NRPS.** The amino acid sequences of the NRPS genes were aligned using Clustal Omega. (A) Additional condensation site region of AU13965. (B) Region of the thiolation site absent in strain AU13965.

(A)

(B)
