# Supplementary material for: Genome comparison between clinical and environmental strains of Herbaspirillum seropedicae reveals a potential new emerging bacterium adapted to human hosts
Source: BMC Genomics. 2019 Aug 2;20:630. doi: 10.1186/s12864-019-5982-9 (PMC6679464; doi:10.1186/s12864-019-5982-9)
Supplement: Supplementary file 6 — Figure S4. Functional classification of Herbaspirillum strain genomes. Functional classification of proteins from clinical and environmental strains was made according to the KEGG categories through the algorithm BlastKOALA. The number of CDSs in each category was normalized by the total number of CDSs in each strain. (DOCX 69 kb) [file 12864_2019_5982_MOESM6_ESM.docx]

**Additional file 6:**

**Figure S4: Functional classification of *Herbaspirillum* strain genomes.** Functional classification of proteins from clinical and environmental strains was made according to the KEGG categories through the algorithm BlastKOALA. The number of CDSs in each category was normalized by the total number of CDSs in each strain.
