# Supplementary material for: Genome comparison between clinical and environmental strains of Herbaspirillum seropedicae reveals a potential new emerging bacterium adapted to human hosts
Source: BMC Genomics. 2019 Aug 2;20:630. doi: 10.1186/s12864-019-5982-9 (PMC6679464; doi:10.1186/s12864-019-5982-9)
Supplement: Supplementary file 7 — Figure S5. Identification of orthologous proteins in H. seropedicae strains. Venn diagram of orthologous proteins between clinical and environmental strains of Herbaspirillum seropedicae. The theoretical proteomes of all strains were compared to each other using blastp. From the blastp results, SRV was calculated. The master SRV cutoff used for clustering was 33. The analysis was performed with the EDGAR software platform. (DOCX 194 kb) [file 12864_2019_5982_MOESM7_ESM.docx]

**Additional file 7:**

**Figure S5: Identification of orthologous proteins in *H. seropedicae* strains.** Venn diagram of orthologous proteins between clinical and environmental strains of *Herbaspirillum seropedicae*. The theoretical proteomes of all strains were compared to each other using blastp. From the blastp results, SRV was calculated. The master SRV cutoff used for clustering was 33. The analysis was performed with the EDGAR software platform.
