# Supplementary material for: Genome comparison between clinical and environmental strains of Herbaspirillum seropedicae reveals a potential new emerging bacterium adapted to human hosts
Source: BMC Genomics. 2019 Aug 2;20:630. doi: 10.1186/s12864-019-5982-9 (PMC6679464; doi:10.1186/s12864-019-5982-9)
Supplement: Supplementary file 8 — Table S3. Comparison of the theoretical proteome of H. seropedicae strains with the ResFam antibiotic resistance gene database. (DOCX 49 kb) [file 12864_2019_5982_MOESM8_ESM.docx]

**Additional file 8:**

**Table S3: Comparison of the theoretical proteome of *H. seropedicae* strains with the ResFam antibiotic resistance gene database.**
