# Supplementary material for: Genome comparison between clinical and environmental strains of Herbaspirillum seropedicae reveals a potential new emerging bacterium adapted to human hosts
Source: BMC Genomics. 2019 Aug 2;20:630. doi: 10.1186/s12864-019-5982-9 (PMC6679464; doi:10.1186/s12864-019-5982-9)
Supplement: Supplementary file 10 — Table S5. Genomic islands of AU13965 strains predicted as “strong”. (DOCX 73 kb) [file 12864_2019_5982_MOESM10_ESM.docx]

**Additional file 10:**

**Table S5: Genomic islands of AU13965 strains predicted as “strong”.**
