# Supplementary material for: Genome comparison between clinical and environmental strains of Herbaspirillum seropedicae reveals a potential new emerging bacterium adapted to human hosts
Source: BMC Genomics. 2019 Aug 2;20:630. doi: 10.1186/s12864-019-5982-9 (PMC6679464; doi:10.1186/s12864-019-5982-9)
Supplement: Supplementary file 11 — Table S6. Neu5Ac metabolism in Herbaspirillum seropedicae. (DOCX 55 kb) [file 12864_2019_5982_MOESM11_ESM.docx]

**Additional file 11:**

**Table S6: Neu5Ac metabolism in *Herbaspirillum seropedicae*.** The strain AU14040 is the only strain capable of synthesizing Neu5Ac. However, transporters for Neu5Ac were identified in all strains of *H. seropedicae* (identity >95%).
